# Hypothermic Machine Perfusion Allows Safe Delay in Kidney Transplantation After Cold Storage

**DOI:** 10.3390/jcm15062173

**Published:** 2026-03-12

**Authors:** Michal Macech, Tadeusz R. Grochowiecki, Ewa Wojtaszek, Slawomir Nazarewski, Tomasz Glogowski, Andrii Mondryk, Michal S. Proczka, Milena N. Michalska, Jolanta Malyszko, Zbigniew Galazka

**Affiliations:** 1 Department of General, Vascular, Endocrine and Transplant Surgery, Medical University of Warsaw, 02-097 Warsaw, Poland; 2 Department of Nephrology, Dialysis and Internal Diseases, Medical University of Warsaw, 02-097 Warsaw, Poland

**Keywords:** kidney transplantation, machine perfusion, delayed graft function

## Abstract

**Background/Objectives**: Static cold storage (SCS) remains the standard method of kidney preservation. As a referral transplant center, we frequently receive kidneys initially preserved with SCS and subsequently initiate prolonged hypothermic machine perfusion (HMP) to extend allocation time and optimize recipient matching. The clinical impact of this sequential preservation strategy remains incompletely defined. To compare outcomes between kidneys preserved with SCS followed by prolonged HMP (SCS+HMP) and SCS alone. **Methods**: This single-center retrospective study included 200 adult recipients of kidney transplants from brain-dead donors (67 SCS+HMP; 133 SCS). Outcomes were primary graft non-function (PNF), delayed graft function (DGF), patient and death-censored graft survival, and renal function over 24 months. Univariable and multivariable analyses identified predictors of DGF. Propensity score matching was performed to adjust for baseline imbalances. **Results**: In the SCS+HMP group, grafts underwent a median of 244 min of SCS followed by 1300 min of HMP, resulting in longer total cold ischemia time than SCS alone (1545 vs. 1104 min; *p* < 0.001). After matching, 51 pairs (*n* = 102) were analyzed. In the matched cohort, PNF occurred in 2 patients (3.9%) in the SCS+HMP group and 3 patients (5.9%) in the SCS group (*p* = 1.0). DGF occurred less frequently in the SCS+HMP group than in the SCS group (17.6% vs. 39.2%; *p* = 0.027). In multivariable Firth penalized logistic regression, HMP was independently associated with lower odds of DGF (OR 0.34; 95% CI 0.13–0.82). During the 24-month follow-up, patient survival, death-censored graft survival, and creatinine trajectories were comparable between groups. **Conclusions**: Sequential HMP after initial SCS enables extended preservation and was associated with a lower incidence of delayed graft function. This strategy does not compromise patient survival, death-censored graft survival, or renal function at 24 months.

## 1. Introduction

Although static cold storage (SCS) at approximately 4 °C remains the standard method of organ preservation, the past three decades have seen substantial technological advances in machine perfusion [1,2]. Numerous improvements in kidney preservation prior to transplantation have been reported. Studies have demonstrated that hypothermic machine perfusion (HMP) reduces the incidence of delayed graft function [3,4] and improves graft survival as well as creatinine clearance [5].

Furthermore, both continuous and pulsatile perfusion have been shown to protect graft cells from apoptosis [6], attenuate ischemia–reperfusion injury [7,8], and decrease the expression of inflammatory cytokines [9]. Several studies suggest that HMP may safely prolong cold ischemia time (CIT) without adversely affecting graft function [10]. However, most of these investigations were conducted in settings where HMP was initiated at the donor center or applied as end-ischemic machine perfusion [11].

As a referral center, we frequently receive kidneys that have initially been preserved using SCS. To allow time for completion of crossmatch testing, recipient optimization, and logistical coordination prior to transplantation, we routinely place the graft on HMP and typically perform transplantation the following morning.

The aim of this study was to evaluate the impact of short-term SCS followed by prolonged HMP on early and medium-term transplant outcomes.

## 2. Materials and Methods

### 2.1. Study Design and Population

This single-center retrospective cohort study reflects real-world kidney allocation practices in Poland and was conducted at a high-volume referral transplant center. The study included 200 consecutive adult patients who underwent kidney transplantation at the Department of General, Vascular, Endocrine and Transplant Surgery, Medical University of Warsaw, between January 2020 and December 2024.

The study protocol was approved by the Local Ethics Committee (AKBE/267/2025) and conducted in accordance with the Declaration of Helsinki and the Declaration of Istanbul.

All transplanted kidneys were procured from donation after brain death (DBD) donors. Detailed donor characteristics are provided in Table 1.

### 2.2. Preservation Protocols and Study Groups

Kidneys undergoing HMP were preserved using the LifePort^®^ Kidney Transporter (Organ Recovery Systems, Chicago, IL, USA) with PumpProtect^®^ preservation solution (Carnamedica, Warsaw, Poland). Static cold storage was performed using StoreProtect Plus^®^ preservation solution (Carnamedica, Warsaw, Poland).

Recipients were stratified according to graft preservation strategy. The SCS+HMP group consisted of 67 recipients who received kidneys initially preserved by SCS followed by HMP, whereas the SCS group included 133 recipients transplanted with kidneys preserved by SCS alone.

**Figure 1:**
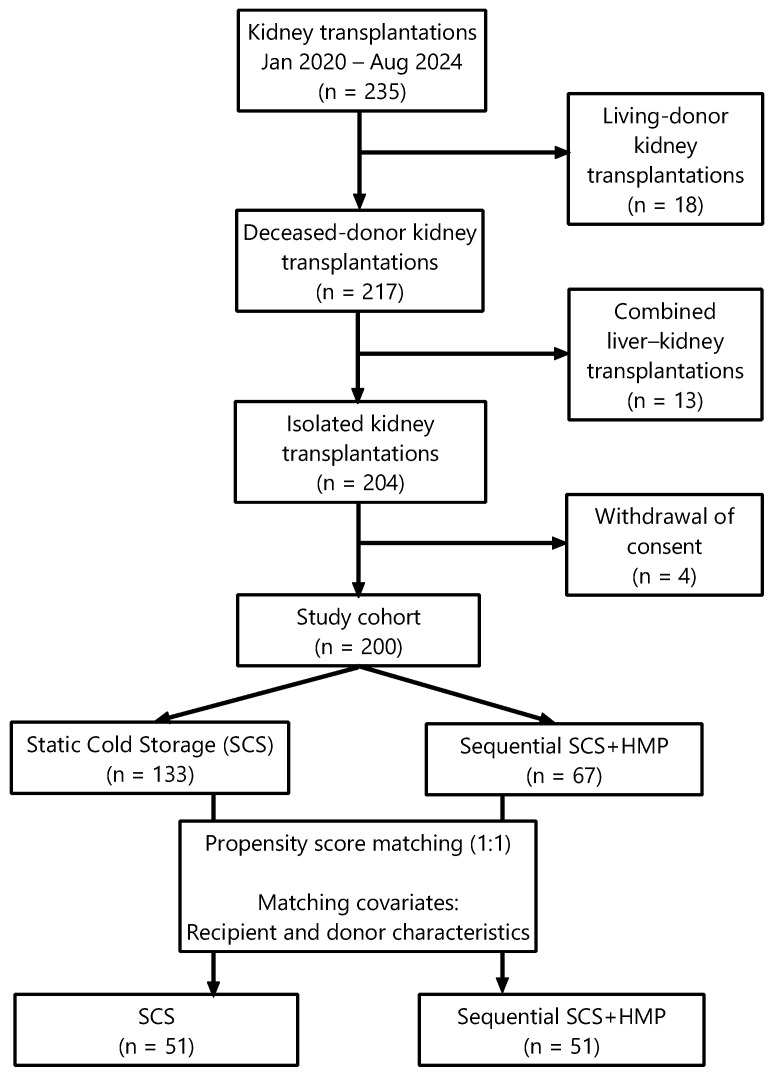
Flow diagram of patient selection and propensity score matching. Abbreviations: SCS—static cold storage; HMP—hypothermic machine perfusion.

Recipient demographic and clinical data were retrospectively collected from institutional records. The study design, patient selection process, and propensity score matching are illustrated in Figure 1.

### 2.3. Immunosuppression

All recipients received a standardized maintenance immunosuppressive regimen consisting of tacrolimus, mycophenolate mofetil, and corticosteroids. Induction therapy with basiliximab or antithymocyte globulin was administered according to individual immunological risk.

### 2.4. Outcomes and Statistical Analysis

Outcomes of interest included delayed graft function (DGF), primary graft non-function (PNF), patient survival, death-censored graft survival, and renal function assessed by serum creatinine at discharge and at 3, 6, 12, and 24 months after transplantation.

#### 2.4.1. Propensity Score Matching

To reduce selection bias and baseline imbalances between preservation groups, propensity score matching (PSM) was performed. Propensity scores were estimated using logistic regression including baseline donor characteristics (Table 1) and selected clinically relevant recipient variables considered potential confounders. Recipient covariates included recipient age, pretransplant dialysis duration, primary cause of end-stage renal disease, diabetes mellitus, coronary artery disease, heart failure, and recipient body mass index.

Cold ischemia time (CIT) was intentionally excluded from the propensity score model because it was considered a post-exposure variable and a potential mediator of the preservation strategy rather than a baseline confounder. CIT is partly determined by the preservation approach itself, particularly in the SCS+HMP group, where prolonged preservation represents an inherent component of the management pathway. Inclusion of CIT in the propensity score model could therefore result in overadjustment and attenuation of the estimated effect of the preservation strategy.

Recipients in the SCS+HMP group were matched 1:1 to SCS recipients using nearest-neighbor matching without replacement on the logit of the propensity score, with a caliper width of 0.2 times the standard deviation of the logit. Covariate balance was assessed using standardized mean differences (SMDs).

#### 2.4.2. Other Analyses

Univariable and multivariable (Firth penalized) logistic regression analyses were performed to identify factors associated with delayed graft function (DGF). Multivariable models included preservation strategy, donor age, ECD status, and donor terminal serum creatinine. Results are presented as odds ratios (ORs) with 95% confidence intervals (CIs).

Patient survival was estimated using the Kaplan–Meier method and compared between groups using the log-rank test. Graft survival was evaluated using (i) a cause-specific Cox proportional hazards model with death treated as a censoring event and (ii) a competing-risk approach based on the Fine–Gray subdistribution hazards model. Hazard ratios (HRs) with corresponding 95% confidence intervals (CIs) are reported. Additionally, restricted mean survival time (RMST) was calculated over the 24-month follow-up period.

Statistical analyses were performed using Statistica version 13.3 (TIBCO Software Inc., Palo Alto, CA, USA) and R version 4.5.2 (R Foundation for Statistical Computing, Vienna, Austria). A *p* value < 0.05 was considered statistically significant.

## 3. Results

A total of 200 kidney transplant recipients were included in the study. Baseline recipient characteristics are presented in Table 2. Details of preservation strategy and ischemia times are summarized in Table 3.

### 3.1. Risk Factors for Delayed Graft Function

Univariate logistic regression analysis was performed to identify donor- and recipient-related risk factors for delayed graft function (DGF) in the entire study cohort (*n* = 200). Only variables demonstrating statistically significant associations are presented in Table 4. Recipient dialysis duration, coronary artery disease, and heart failure, as well as donor age, terminal donor serum creatinine, and expanded criteria donor (ECD) status, were significantly associated with an increased risk of DGF.

### 3.2. Propensity Score-Matched Analysis

Propensity score matching yielded 51 well-balanced pairs (*n* = 102). Covariate balance after matching was adequate, with all standardized mean differences <0.12 (Supplementary Table S1; Supplementary Figure S1).

In the matched cohort, hypothermic machine perfusion was associated with a lower odds of delayed graft function in multivariable Firth penalized logistic regression analysis (OR 0.34; 95% CI 0.13–0.82).

Delayed graft function occurred in 9 of 51 recipients (17.6%) in the SCS+HMP group and 20 of 51 recipients (39.2%) in the SCS group; *p* = 0.027 (absolute difference, 21.6 percentage points; 95% CI 4.0–39.1 percentage points). The incidence of primary graft non-function was comparable between groups (SCS+HMP: 2 patients [3.9%] vs. SCS: 3 patients [5.9%]; *p* = 1.0).

### 3.3. Survival Outcomes

**Figure 2:**
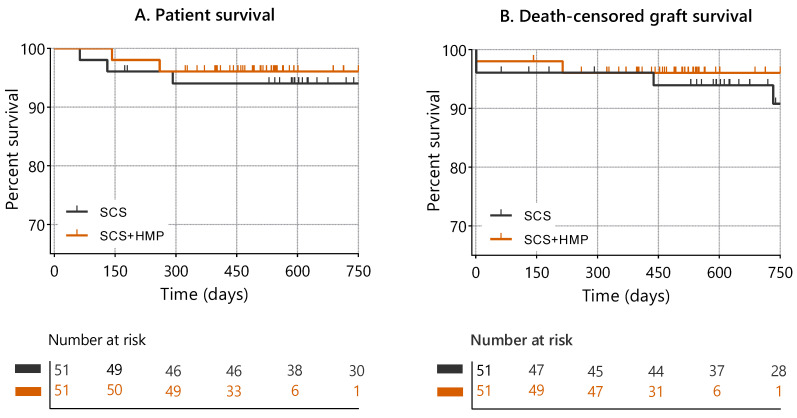
Kaplan–Meier curves for patient and death-censored graft survival in the propensity score-matched cohort (*n* = 51 per group). Patient survival (**panel A**) and death-censored graft survival (**panel B**) are shown over 24 months. Tick marks indicate censored observations. Comparisons between preservation strategies were performed using the log-rank test (patient survival *p* = 0.63; death-censored graft survival *p* = 0.68).

Cumulative patient survival and death-censored graft survival did not differ significantly between preservation strategies (log-rank *p* = 0.63 and *p* = 0.68, respectively) (Figure 2). Survival probabilities at 3, 6, 12, and 24 months are presented in Table 5.

In Cox proportional hazards regression analysis, preservation strategy was not significantly associated with patient survival (HR 0.66; 95% CI 0.19–2.35; *p* = 0.52).

For death-censored graft survival, cause-specific Cox regression similarly showed no significant association between preservation strategy and graft failure (HR 0.69; 95% CI 0.12–4.08; *p* = 0.68).

In competing-risk analysis using the Fine–Gray subdistribution hazard model, hypothermic machine perfusion was likewise not significantly associated with graft failure when death was treated as a competing event (sHR 0.65; 95% CI 0.11–3.70 *p* = 0.62).

Restricted mean survival time (RMST) analysis over 24 months demonstrated no statistically significant differences between groups. The RMST for graft survival was 725 days in the SCS+HMP group versus 713 days in the SCS group (absolute difference, 11.2 days; 95% CI −43.1 to 65.5; *p* = 0.69). For patient survival, the RMST was 703 days versus 679 days, respectively (absolute difference, 24.2 days; 95% CI −46.6 to 94.9; *p* = 0.50).

### 3.4. Renal Function and Ischemia Time Analyses

**Figure 3:**
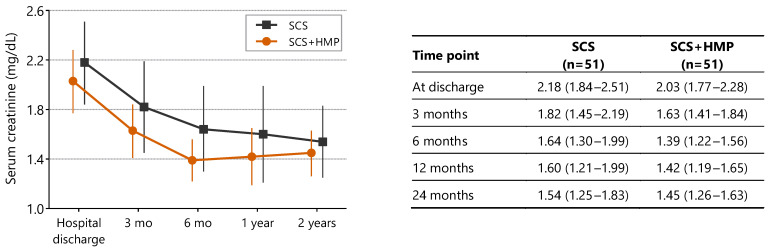
Serum creatinine levels during follow-up (*n* = 51 per group). Values are presented as mean ± 95% confidence interval. Renal function improved over time in both groups, with no significant difference between preservation strategies (repeated-measures ANOVA, *p* = 0.19). Abbreviations: SCS—static cold storage; HMP—hypothermic machine perfusion.

Serum creatinine levels did not differ significantly between preservation strategies over the 24-month follow-up (repeated-measures ANOVA, *p* = 0.19). In both groups, serum creatinine levels improved significantly within the first 6 months after transplantation compared with discharge values (*p* < 0.001; Figure 3).

Cold ischemia time (CIT) was significantly longer in the SCS+HMP group (1545 min; 95% CI 1431–1660) than in the SCS group (1104 min; 95% CI 1008–1200; *p* < 0.001). After propensity score matching, CIT remained longer in the SCS+HMP group (1516 vs. 1105 min; *p* = 0.005), reflecting intrinsic characteristics of the preservation strategies. The distribution of CIT before and after matching is shown in Supplementary Figure S2.

In multivariable analysis, CIT analyzed as a continuous variable was not independently associated with DGF (OR per minute 1.0001; 95% CI 0.9995–1.0008; *p* = 0.68).

Similarly, hypothermic machine perfusion duration was not significantly associated with DGF when analyzed either as a continuous variable or according to quartiles.

## 4. Discussion

This study evaluated the clinical impact of a sequential kidney graft preservation strategy consisting of short static cold storage followed by prolonged hypothermic machine perfusion (SCS+HMP), compared with static cold storage (SCS) alone in a tertiary referral-center setting. The key finding is that sequential preservation was associated with a lower incidence of delayed graft function. Importantly, this effect was observed despite longer total preservation time in the SCS+HMP group.

Delayed graft function remains one of the most relevant early complications after kidney transplantation. Even short-term dysfunction may influence hospital stay, immunosuppressive management, and early graft assessment. Therefore, strategies that reduce DGF are clinically meaningful.

Static cold storage, which is regarded as the standard preservation method, reduces metabolic demand but does not provide active metabolic support. Even under hypothermic conditions, residual cellular metabolism persists, leading to depletion of energy stores, disruption of ion homeostasis, and accumulation of metabolic byproducts [12]. In addition, cessation of flow during static storage promotes endothelial dysfunction and increases susceptibility to ischemia–reperfusion injury [13,14]. In contrast, hypothermic machine perfusion maintains continuous circulation of preservation solution, supports microvascular flow, and facilitates removal of metabolic waste. Experimental and clinical studies suggest that this may limit ischemic injury and improve early graft function [15,16]. Consistent with the above mechanisms, we observed a 66% reduction in the odds of delayed graft function in our cohort (odds ratio, 0.34).

Notably, the benefit in early graft function occurred even though total cold ischemia time was substantially longer in the SCS+HMP group. Traditionally, prolonged preservation time is considered a major risk factor for DGF [17,18,19]. Our findings suggest that the method of preservation may be at least as important as its duration. When dynamic perfusion is applied, longer preservation time does not necessarily translate into worse early outcomes.

Importantly, the benefit observed in early graft function did not come at the expense of later outcomes. Patient survival, death-censored graft survival, and longitudinal renal function were comparable between groups. These findings indicate that extending preservation time in combination with hypothermic machine perfusion does not compromise overall transplant outcomes.

At the time of its introduction at our center, the sequential preservation strategy was adopted primarily for logistical reasons. During the COVID-19 pandemic, national transplant policy required recipients to undergo COVID-19 testing and high-resolution chest computed tomography prior to transplantation. In approximately 20% of cases, newly detected abnormalities led to recipient disqualification and necessitated additional time for recipient reassignment [20], thereby prolonging the pre-implantation interval. In this setting, sequential static cold storage (SCS) followed by hypothermic machine perfusion (HMP) provided a practical solution to maintain graft viability during unavoidable delays. Over time, however, the rationale for this approach evolved. HMP was increasingly applied not only to facilitate scheduling but also to improve graft condition prior to implantation, unless technical constraints prevented its use (e.g., inability to cannulate the renal artery) or the anticipated time to revascularization was less than 6 h. The present findings support this evolution in practice.

The preservation protocol evaluated here differs from strategies predominantly investigated in randomized controlled trials and more closely reflects real-world practice. Most trials have assessed continuous HMP initiated at the donor center [3,4,21,22,23] or short end-ischemic perfusion [24,25,26]. In contrast, our study examined prolonged HMP initiated after an initial phase of SCS, typically after organ arrival at a referral transplant center.

Preservation approaches similar to ours have been described. Adani et al. reported that delayed initiation of HMP after static cold storage may improve graft hemodynamics and attenuate ischemic injury accumulated during SCS [27]. Likewise, Patel et al. evaluated donation after circulatory death kidneys undergoing HMP following static cold storage and reported a reduction in delayed graft function from approximately 39% with SCS alone to 28% with sequential HMP, corresponding to a relative reduction of about 30% [28]. Importantly, this benefit was achieved without adverse effects on graft survival. These data provide quantitative support for the concept that initiation of machine perfusion after transportation may still confer measurable clinical benefit and are directionally consistent with our findings.

Several limitations should, however, be acknowledged. As a retrospective observational study, treatment allocation was not randomized, introducing potential selection bias and logistic confounding. Kidneys assigned to HMP were selected based on clinical judgment and anticipated logistical delays. Although propensity score matching reduced imbalances in measured variables, residual confounding from unmeasured factors cannot be excluded. An additional consideration pertains to the number-at-risk distributions in the Kaplan–Meier analyses. A marked decline in the SCS+HMP group beyond the first post-transplant year reflects administrative censoring rather than an excess of events. Because SCS+HMP became routine practice in our clinic from 2023 onward and the database was closed in 2025, many patients in this group had not yet reached two years of follow-up at the time of analysis. In contrast, most patients in the SCS group were transplanted earlier and therefore had longer available follow-up. Accordingly, the comparison partly reflects a newer institutional standard versus an earlier treatment period, and survival estimates at later time points in the SCS+HMP group should be interpreted with appropriate caution.

Despite these limitations, the study has notable strengths. The cohort reflects contemporary transplant activity in a high-volume tertiary referral center managing marginal donors and medically complex recipients. The preservation strategy evaluated addresses practical logistical challenges encountered in routine transplantation. Furthermore, clinically meaningful endpoints—including DGF, primary non-function, survival, and longitudinal renal function—were systematically assessed.

In summary, sequential preservation using static cold storage followed by prolonged hypothermic machine perfusion reduces the incidence of delayed graft function. Importantly, this improvement in early graft performance occurs despite extended preservation time and without compromising patient survival, graft survival, or renal function, supporting its role in contemporary transplant practice.

## Figures and Tables

**Table 1:** Donor characteristics.

	SCS+HMP *n* = 67	SCS *n* = 133	*p* Value
Age (years)	49 (42–59)	45 (36–53)	0.08
Terminal serum creatinine (mg/dL)	2.31 ± 1.86	1.41 ± 1.01	0.001
Urine output, 24 h pre-procurement (mL)	2647 ± 1264	3402 ± 1503	<0.001
Cardiac arrest before procurement	17 (25.4%)	27 (20.3%)	0.47
Expanded criteria donor	18 (26.9%)	10 (7.5%)	<0.001

Data are presented as mean ± standard deviation (SD), median (interquartile range, IQR), or number (percentage), as appropriate. Abbreviations: SCS—static cold storage; HMP—hypothermic machine perfusion.

**Table 2:** Baseline recipient characteristics.

	SCS+HMP *n* = 67	SCS *n* = 133	*p* Value
Age (years)	52 (25–62)	50 (41–61)	0.42
Primary cause of end-stage renal disease			
Diabetic nephropathy	3 (4.5%)	9 (6.8%)	0.75
Glomerulonephritis	22 (32.9%)	49 (36.8%)	0.64
Hypertensive nephropathy	5 (8.1%)	11 (8.3%)	1.0
Interstitial nephropathy	2 (3%)	8 (6%)	0.50
Polycystic kidney disease	10 (14.9%)	25 (18.8%)	0.56
Other	9 (13.4%)	14 (10.5%)	0.64
Unknown	16 (23.9%)	17 (12.7%)	0.07
Dialysis duration before KTx (months)	36.3 (15–48)	35.7 (12–45)	0.84
Recipient comorbidities			
Diabetes mellitus	9 (13.4%)	14 (10.5%)	0.64
Hypertension	66 (98.5%)	127 (95.4%)	0.42
Coronary artery disease	15 (22.4%)	25 (18.8%)	0.58
Heart failure	29 (43.3%)	22 (16.5%)	<0.001
Stroke	2 (3%)	4 (3%)	1.0
Pulmonary disease	4 (6%)	8 (6%)	1.0
History of malignancy	5 (7.5%)	10 (7.5%)	1.0
Body mass index at transplantation (kg/m^2^)	26.1 (24–28.4)	24.7 (21.8–27.3)	0.02
PRA, median (min–max)	0 (0–65)	0 (0–86)	0.54
HLA mismatch			
0	2 (3%)	2 (3%)	1
1	3 (4.5%)	9 (6.8%)	0.75
2	18 (26.9%)	22 (16.5%)	0.09
3	21 (31.3%)	49 (36.8%)	0.53
4	9 (13.4%)	31 (23.3%)	0.07
5	12 (17.9%)	15 (11.3%)	0.19
6	6 (8.9%)	6 (4.5%)	0.22
Induction immunosuppression			
None	49 (73.1%)	82 (61.6%)	0.11
Basiliximab	16 (23.9%)	43 (32.3%)	0.25
Antithymocyte globulin	2 (3%)	8 (6%)	0.49
Transplant sequence			
First transplant	56 (83.4%)	117 (87.9%)	0.38
Second transplant	11 (16.4%)	11 (8.3%)	0.09
Third transplant	0	4 (3%)	0.30

Data are presented as mean ± standard deviation (SD), median (interquartile range, IQR), or number (percentage), as appropriate. Abbreviations: SCS—static cold storage; HMP—hypothermic machine perfusion; KTx—kidney transplantation, PRA—panel reactive antibody; HLA—human leukocyte antigen.

**Table 3:** Preservation time components (minutes) before and after propensity score matching.

	Before Propensity Score Matching		After Propensity Score Matching	
	SCS+HMP (*n* = 67)	SCS (*n* = 133)	SCS+HMP (*n* = 51)	SCS (*n* = 51)
SCS duration	244 (106–342)	1104 (711–1467)	222 (104–281)	1105 (707–1574)
HMP duration	1300 (1020–1600)	N/A	1309 (950–1600)	N/A
Total CIT	1545 (1173–1883)	1104 (711–1467)	1516 (1159–1778)	1105 (707–1574)

Values are presented as median (interquartile range, IQR). Preservation times reflect intrinsic characteristics of preservation strategies. Total cold ischemia time (CIT) differed significantly between groups both before matching (*p* < 0.001) and after matching (*p* < 0.001). CIT was not included in the propensity score model, as it was considered a post-exposure variable related to the preservation strategy.

**Table 4:** Univariate analysis of risk factors for delayed graft function in the study cohort (*n* = 200).

Variable	Exposure Contrast	OR	95% CI	*p* Value
Recipient				
Dialysis duration	Per month increase	1.01	1.001–1.018	0.025
Coronary artery disease	Present	2.62	1.28–5.37	0.008
Heart failure	Present	2.49	1.28–4.83	0.007
Donor				
Age	Per year increase	1.02	1.001–1.048	0.041
Terminal serum creatinine	Per 1 mg/dL increase	1.37	1.11–1.69	0.003
Expanded criteria donor	Yes	2.74	1.21–6.18	0.015

Abbreviations: OR—odds ratio; CI—confidence interval.

**Table 5:** Cumulative patient and death-censored graft survival at 24 months according to preservation strategy in the propensity-matched cohort.

	Patient Survival		Death-Censored Graft Survival	
Time Post-Transplant	SCS+HMP (*n* = 51)	SCS (*n* = 51)	SCS+HMP (*n* = 51)	SCS (*n* = 51)
3 months	100% (100–100)	98% (94–100)	98% (94–100)	96% (91–100)
6 months	98% (94–100)	96% (91–100)	98% (94–100)	96% (91–100)
12 months	96% (91–100)	94% (87–100)	96% (91–100)	96% (91–100)
24 months	96% (91–100)	94% (87–100)	96% (91–100)	94% (88–100)

Values represent Kaplan–Meier estimated cumulative survival probabilities; 95% confidence intervals (CIs) are provided in parentheses. Abbreviations: SCS—static cold storage; HMP—hypothermic machine perfusion.

## Data Availability

The data presented in this study are available on request from the corresponding author.

## Supplementary Materials

The following supporting information can be downloaded at: https://www.mdpi.com/article/10.3390/jcm15062173/s1, Table S1: Covariate balance before and after propensity score matching. Baseline characteristics included in the propensity score model are presented for the SCS and SCS+HMP groups before and after matching. Standardized mean differences (SMD) were calculated to quantify between-group imbalance, with values < 0.1 considered indicative of adequate covariate balance. Figure S1: Absolute standardized mean differences before and after propensity score matching. Love plot displaying absolute standardized mean differences (SMDs) for donor and recipient variables included in the propensity score model. Each point represents the absolute SMD for an individual variable. The vertical reference line at 0.1 indicates the prespecified threshold for acceptable balance. After matching, all covariates exhibited SMDs below 0.1, indicating adequate balance between groups. Figure S2: Distribution of cold ischemia time before and after propensity score matching. Panel (A) presents the distribution of CIT in the unmatched cohort (SCS+HMP: *n* = 67; SCS: *n* = 133). Panel (B) shows the distribution after 1:1 propensity score matching (*n* = 51 per group). CIT remained longer in the SCS+HMP group both before and after matching.

## Abbreviations

The following abbreviations are used in this manuscript:

ANOVA	Analysis of Variance
BMI	Body Mass Index
CI	Confidence Interval
CIT	Cold Ischemia Time
DBD	Donation after Brain Death
DGF	Delayed Graft Function
ECD	Expanded Criteria Donor
HLA	Human Leukocyte Antigen
HMP	Hypothermic Machine Perfusion
HR	Hazard Ratio
IQR	Interquartile Range
KTx	Kidney Transplantation
OR	Odds Ratio
PNF	Primary Non-Function
PRA	Panel Reactive Antibody
PSM	Propensity Score Matching
RMST	Restricted Mean Survival Time
sHR	Subdistribution Hazard Ratio
SCS	Static Cold Storage
SD	Standard Deviation
SMD	Standardized Mean Difference
