# Supplementary material for: Hypothermic Machine Perfusion Allows Safe Delay in Kidney Transplantation After Cold Storage
Source: J Clin Med. 2026 Mar 12;15(6):2173. doi: 10.3390/jcm15062173 (PMC13026752; doi:10.3390/jcm15062173)
Supplement: Supplementary file 1 [file jcm-15-02173-s001.zip › Supplementary Figure S1.pdf]

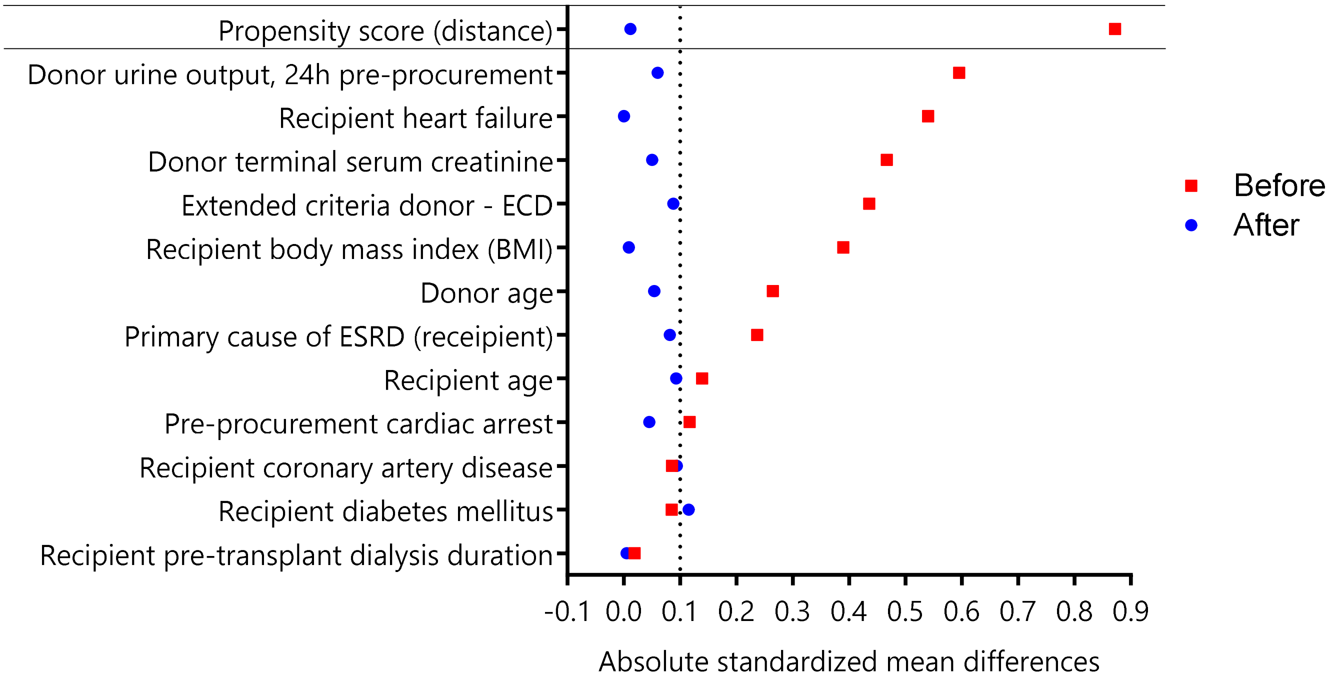

Figure S1. Absolute standardized mean differences before and after propensity score matching. Love plot displaying absolute standardized mean differences (SMDs) for donor and recipient variables included in the propensity score model. Each point represents the absolute SMD for an individual variable. The vertical reference line at 0.1 indicates the prespecified threshold for acceptable balance. After matching, all covariates exhibited SMDs below 0.1, indicating adequate balance between groups.
