# Supplementary material for: Hypothermic Machine Perfusion Allows Safe Delay in Kidney Transplantation After Cold Storage
Source: J Clin Med. 2026 Mar 12;15(6):2173. doi: 10.3390/jcm15062173 (PMC13026752; doi:10.3390/jcm15062173)
Supplement: Supplementary file 1 [file jcm-15-02173-s001.zip › Supplementary Figure S2.pdf]

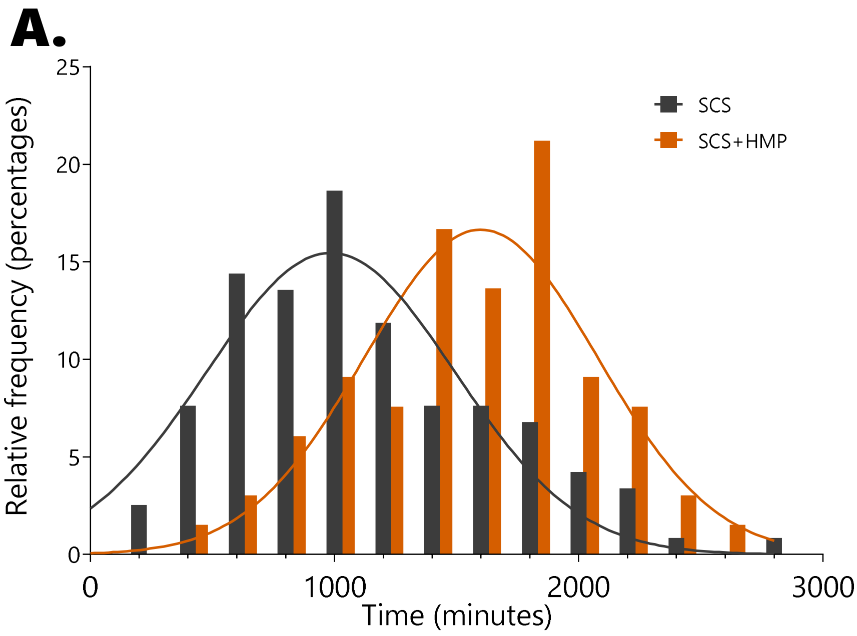

	SCS n=133	SCS +HMP n=67
Minimum	181	444
25% Percentile	711	1173
Median	1016	1580
75% Percentile	1467	1883
Maximum	2700	2595
Mean	1104	1545
Std. Deviation	527	466
Std. Error of Mean	48	57
Lower 95% CI of mean	1008	1430
Upper 95% CI of mean	1200	1659

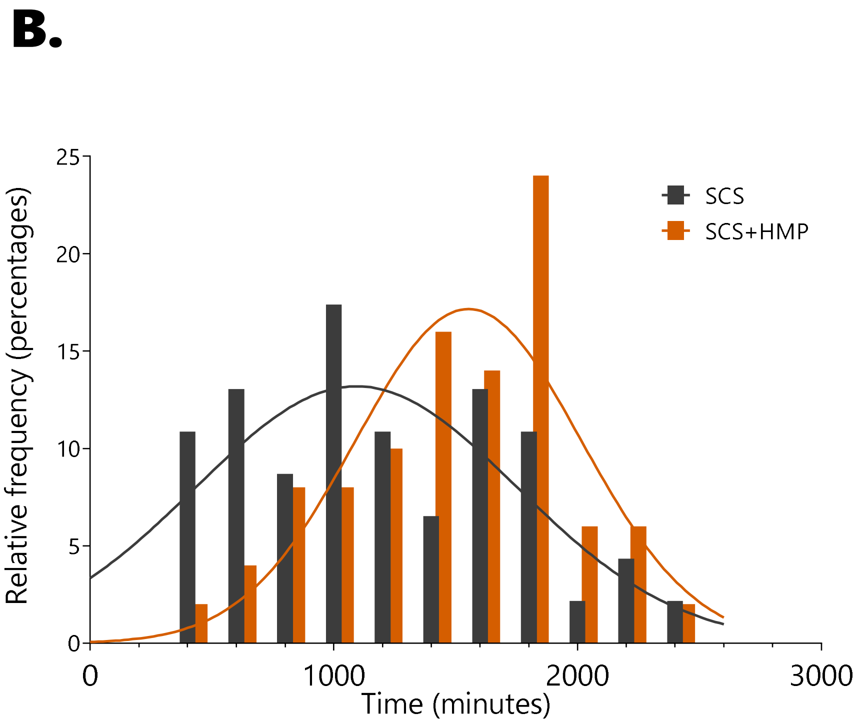

	SCS n=51	SCS +HMP n=51
Minimum	320	469
25% Percentile	707	1159
Median	1105	1516
75% Percentile	1574	1778
Maximum	2340	2470
Mean	1178	1480
Std. Deviation	541	455
Std. Error of Mean	79.7	64.3
Lower 95% CI of mean	1017	1351
Upper 95% CI of mean	1338	1609

Figure S2. Distribution of cold ischemia time before and after propensity score matching. Panel (A) presents the distribution of CIT in the unmatched cohort (SCS+HMP: n = 67; SCS: n = 133). Panel (B) shows the distribution after 1:1 propensity score matching (n = 51 per group). CIT remained longer in the SCS+HMP group both before and after matching.
