# Supplementary material for: Hypothermic Machine Perfusion Allows Safe Delay in Kidney Transplantation After Cold Storage
Source: J Clin Med. 2026 Mar 12;15(6):2173. doi: 10.3390/jcm15062173 (PMC13026752; doi:10.3390/jcm15062173)
Supplement: Supplementary file 1 [file jcm-15-02173-s001.zip › Supplementary Table S1.pdf]

**Table S1.** Covariate balance before and after propensity score matching. Baseline characteristics included in the propensity score model are presented for the SCS and SCS+HMP groups before and after matching. Standardized mean differences (SMD) were calculated to quantify between-group imbalance, with values <0.1 considered indicative of adequate covariate balance.

| | **Before** | **After** |
|---|---|---|
| Propensity score (distance) | 0.872 | 0.012 |
| Donor urine output, 24h pre-procurement | 0.595 | 0.06 |
| Recipient heart failure | 0.54 | 0 |
| Donor terminal serum creatinine | 0.467 | 0.05 |
| Extended criteria donor - ECD | 0.436 | 0.088 |
| Recipient body mass index (BMI) | 0.39 | 0.009 |
| Donor age | 0.265 | 0.054 |
| Primary cause of ESRD (receipient) | 0.237 | 0.082 |
| Recipient age | 0.139 | 0.093 |
| Pre-procurement cardiac arrest | 0.117 | 0.045 |
| Recipient coronary artery disease | 0.086 | 0.094 |
| Recipient diabetes mellitus | 0.085 | 0.115 |
| Recipient pre-transplant dialysis duration | 0.019 | 0.005 |

Abbreviations: SCS – static cold storage; HMP – hypothermic machine perfusion.
